# Congenital upper eyelids ectropion in Down’s syndrome

**DOI:** 10.3205/oc000054

**Published:** 2017-02-03

**Authors:** Rafael Corredor-Osorio, José Luis Tovilla-Pomar, José Luis Tovilla-Canales

**Affiliations:** 1 Department of Oculoplastic and Orbit, Institute of Ophthalmology, “Conde de Valenciana”, México City, México

**Keywords:** Down’s syndrome, congenital ectropion, skin grafts, eyelid

## Abstract

Congenital bilateral ectropion of the upper eyelids is a rare, benign condition reported in ophthalmic literature. It is more frequently associated with Down’s syndrome, ichthyosis, and sporadic cases in newborns from black population. We report three cases of congenital bilateral upper eyelid ectropion associated with Down’s syndrome. Management of these patients usually requires medial and lateral canthoplasties, full-thickness pentagonal resection of the upper eyelids and placement of skin grafts. We present herein the evolution of one of these patients and we will discuss the mechanism of the eyelid ectropion and its treatment.

## Introduction

Congenital bilateral ectropion of the upper eyelids is a rare, benign condition [1], [2], [3]. The eversion usually presents at birth and resolves spontaneously within two weeks of birth [1], [3], [4]. Its etiology is unknown and several possible mechanisms have been proposed, however it is frequently associated with Down’s syndrome, ichthyosis, and newborns in the black population [1], [2], [4]. We present three cases of this very rare condition and we will discuss the mechanism of the eyelid ectropion and its management.

## Case descriptions

### Case 1 

**Figure 1:**
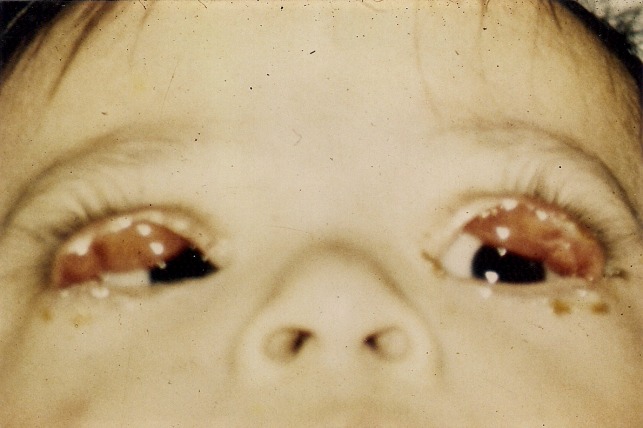
Age: 8 months. Preoperative picture showing marked bilateral, congenital ectropion of the upper eyelids

An eight-month-old boy with clinical signs and diagnosis of Down’s syndrome was brought to consultation because of bilateral ectropion of the upper eyelids that was noted at birth (Figure 1 (Fig. 1)). He was born at term to a 33-year-old mother following an uneventful pregnancy and delivery. Chromosome analysis showed trisomy 21 (karyotype 47 xy + 21). Among the typical phenotypic facial changes of Down’s syndrome, he was diagnosed with a congenital heart disease, polydactyly in the left hand and adduct right foot. His ophthalmic examination revealed horizontal nystagmus, strabismus, epicanthal folds and ectropion of both upper eyelids as a result of severe anterior lamella shortening. With a gentle digital pressure the upper eyelids could be repositioned but were everted again while crying or with forced lid closure. The corneas remained clear in both eyes. Visual acuity could not be recorded and the rest of his ophthalmic exam was within normal limits.

Due to the previous findings, the patient was taken to surgery for bilateral correction of the upper eyelid ectropion. A horizontal skin incision was performed 2 mm above the upper eyelid margin and the edges of the wound were undermined and separated. Once the skin was loose, the eyelid returned to its normal position. A severe laxity of both upper eyelids was noted due to canthal laxity that was corrected at this point with a medial and lateral canthopexy using a 5-0 Vicryl suture for bony attachment, and a full-thickness pentagonal resection of 3 mm at the junction of the lateral one-third and the medial two-thirds of the lid. The margins are closed with three 6-0 black silk suture and the tarsus are closed with interrupted 5-0 Vicryl suture. 

**Figure 2:**
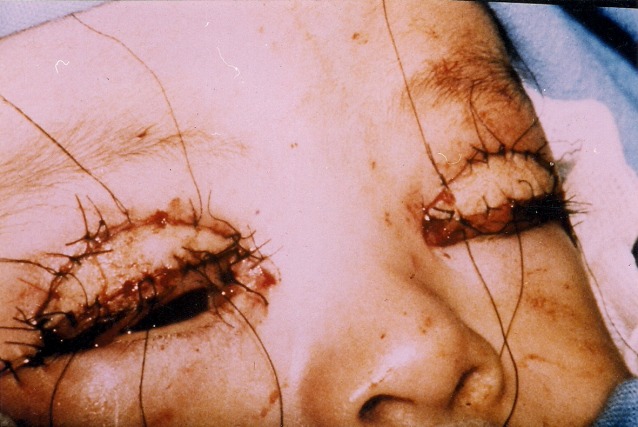
Intraoperative picture showed full-thickness skin graft upper eyelids.

**Figure 3:**
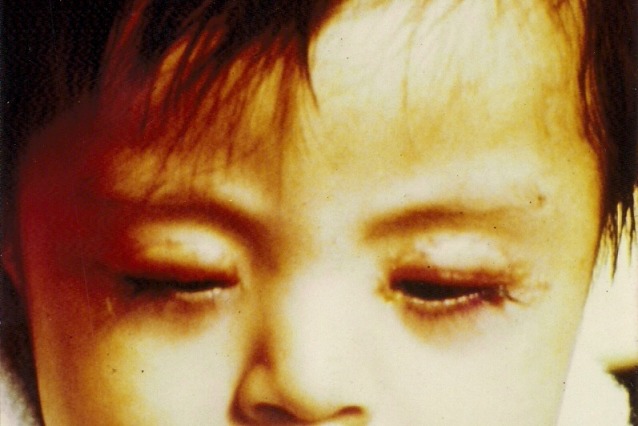
The postoperative result with satisfactorily reformed upper eyelids margins

This helped to improve the horizontal laxity. Finally, two elliptical retroauricular skin grafts measuring 12 mm long and 5 mm wide were grafted into upper eyelids and sutured with interrupted 6/0 black silk suture (Figure 2 (Fig. 2)). Postoperative there were no complications and the final outcome was satisfactory (Figure 3 (Fig. 3)).

### Case 2

**Figure 4:**
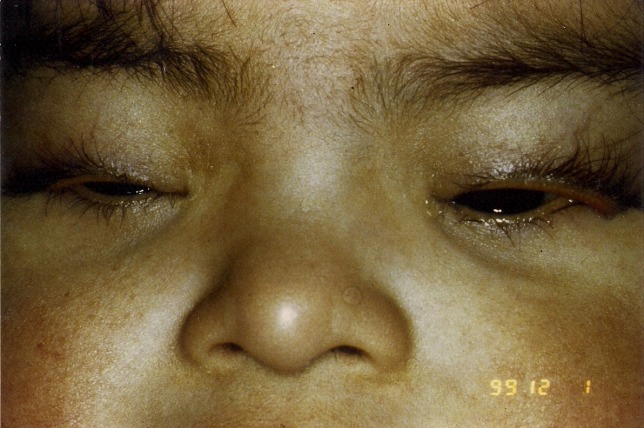
10-month-old child with congenital ectropion of the upper eyelids associated with Down’s syndrome

A 10-month-old boy was brought to consultation because of bilateral ectropion of the upper eyelids since the neonatal period. He was born to a 35-year-old mother following an uncomplicated pregnancy and delivery. Initial management was conservative, consisting of frequent application of topical lubricants and ointments and patching of eyelids. Ocular examination revealed: upward slant of the eyelid fissures, epicanthal folds, and ectropion of both upper eyelids, which were able to be repositioned easily, but returned to the everted position spontaneously with crying and forced closure (Figure 4 (Fig. 4)). Chromosome analysis showed trisomy 21 (karyotype 47, xy + 21). Pediatric evaluation revealed congenital heart disease, cryptorchidism and umbilical herniation. We proposed a surgical correction of both eyelids, but the parents refused surgery and lost follow-up.

### Case 3

**Figure 5:**
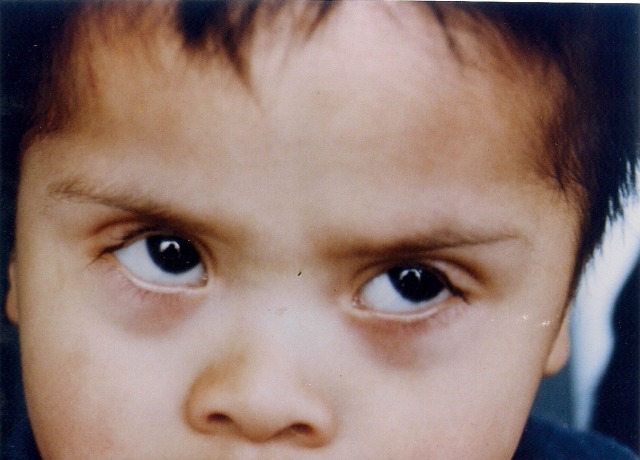
11-month-old child with bilateral total congenital ectropion of the upper eyelids in Down’s syndrome

A boy with Down’s syndrome was first brought to medical attention to us at the age of 11 months because of ectropion of the upper eyelids since birth that accentuated while crying. His 33-year-old mother reported that the pregnancy was uneventful, the labour was not prolonged and delivery was normal vaginal. As with the previous cases, the eyelids could be returned easily to their normal positions but immediately turned out again with force closure (Figure 5 (Fig. 5)). His karyotyping was 47, xy + 21. Both corneas were clear and showed no fluorescein staining. We proposed a surgical correction, but the parents refused surgery and lost follow-up.

## Discussion

Congenital ectropion of the upper eyelids was first described by Adams in 1896 [1], [2], [3], [4]. Later, Gilbert and co-workers described two more cases associated with Down’s syndrome [5], [6]. This rare condition has been reported more frequently in black infants [1], [2], [3], [4], [7], [8] associated with ichthyosis [1], [4], [9], [10] and in infants with trisomy 21 [2], [5], [8], [11], [12], [13], [14]. Although the condition is generally bilateral and asymmetrical, some unilateral cases have been described [8], [11], [13]. Down’s syndrome encompasses numerous ocular abnormalities like myopia, keratoconus, nystagmus, epiblepharon, brushfields spots, hypertelorism, epicanthus, convergent strabismus, cataracts, blepharoconjuctivitis with the epicanthal folds, and the typical mongoloid slant to the eyelid fissures being the most obvious periocular findings [6], [12].

Although the physiopathology of congenital upper eyelid ectropion is unknown, multiple factors have been implied, including absence of an effective lateral canthal ligaments, lateral elongation of the eyelid, hypotonia of the orbicularis, vertical shortening of the anterior lamella, and failure of the orbital septum to fuse with the levator aponeurosis [1], [2], [4], [6], [11], [12], [13], [14].

Treatment of congenital upper eyelid ectropion is controversial. Different options have been suggested. Some believe that a simple and conservative management with lubricants ointments and moist chambers may be enough to prevent desiccation of the exposed conjunctiva, reduction of conjunctival edema and to allow spontaneous inversion of the eyelid within 2 to 3 weeks. [3], [7], [11], [13].

Surgical treatment for more severe cases that did not respond to conservative treatment include sub-conjunctival injection of hyaluronic acid [4], [8], [13], tarsorraphy [2], [3], [6], [7] tarsorraphy with excision of redundant conjunctiva [5], [7], fornix suture [3], [13], full-thickness skin graft [1], [2], [5], [11], full-thickness horizontal lid shortening [2], [6], and attachment of the orbital septum to the levator aponeurosis [2]. Most cases of congenital eversion of the eyelids without Down’s syndrome responded to patching or taping of the eyelids and the use of ointments [3], [6], [7], [11], [13], however surgical intervention may be necessary in patients with Down’s syndrome [6], [12], [14]. In our cases, the subconjunctival hyaluronic acid injection was not available at the moment and it was not likely to be effective due the lack of conjunctival chemosis.

We believe that correct management to the congenital ectropion upper eyelid in these cases should include the correction of the underlying anterior lamella shortening with full-thickness skin grafts that should be extended beyond the horizontal limb of the canthal tendon to compensate for subsequent contraction of the graft. In addition, horizontal lid laxity needs to be addressed with a lateral and medial tarsal strip procedure and a full-thickness pentagonal lid resection. 

In our three cases, the patients did not fulfill the conservative treatment parameters due the congenital abnormalities of the eyelids that may occur in Down’s syndrome.

In conclusion, congenital ectropion of the upper eyelid is a rare abnormality that can threaten the cornea and visual acuity if not treated early. In cases where the eyelids can be repositioned mechanically but continue to evert with forced closure or crying, we recommend surgical intervention to prevent further complications. 

Management’s goal is to protect the cornea, improve cosmesis, and prevent amblyopia. 

## Notes

### Competing interests

The authors declare that they have no competing interests.

### Informed consent 

Written informed consent was obtained from the patients’ parents for the publication of these case reports and accompanying images.
